# Loukoumasomes Are Distinct Subcellular Structures from Rods and Rings and Are Structurally Associated with MAP2 and the Nuclear Envelope in Retinal Cells

**DOI:** 10.1371/journal.pone.0165162

**Published:** 2016-10-31

**Authors:** Jake W. Noble, Diana V. Hunter, Calvin D. Roskelley, Edward K. L. Chan, Julia Mills

**Affiliations:** 1 Department of Biology, Trinity Western University, Langley, British Columbia, Canada; 2 International Collaboration on Repair Discoveries (ICORD), University of British Columbia, Vancouver, British Columbia, Canada; 3 Department of Cellular and Physiological Sciences, University of British Columbia, Vancouver, British Columbia, Canada; 4 Department of Oral Biology, University of Florida, Gainesville, Florida, United States of America; 5 Department of Molecular Biology and Biochemistry, Simon Fraser University, Burnaby, British Columbia, Canada; Institut de Genetique et Developpement de Rennes, FRANCE

## Abstract

“Rods and rings” (RR) and loukoumasomes are similarly shaped, subcellular macromolecular structures with as yet unknown function. RR, so named because of their shape, are formed in response to inhibition in the GTP or CTP synthetic pathways and are highly enriched in the two key enzymes of the nucleotide synthetic pathway. Loukoumasomes also occur as linear and toroidal bodies and were initially inferred to be the same as RR, largely due to their shared shape and size and the fact that it was unclear if they shared the same subcomponents. In human retinoblastoma tissue and cells we have observed toroidal, perinuclear, macromolecular structures of similar size and antigenicity to those previously reported in neurons (neuronal-loukoumasomes). To further characterize the subcomponents of the retinal-loukoumasomes, confocal analysis following immunocytochemical staining for alpha-tubulin, beta-III tubulin and detyrosinated tubulin was performed. These studies indicate that retinal-loukoumasomes are enriched for beta-III tubulin and other tubulins associated with microtubules. Immunofluorescence together with the in situ proximity ligation assay (PLA), confirmed that beta-III tubulin colocalized with detyrosinated tubulin within loukoumasomes. Our results indicate that these tissues contain only loukoumasomes because these macromolecular structures are immunoreactive with an anti-tubulin antibody but are not recognized by the prototype anti-RR/inosine monophosphate dehydrogenase (IMPDH) antibody (It2006). To further compare the RR and retinal-loukoumasomes, retinoblastoma cells were exposed to the IMPDH-inhibitor ribavirin, a drug known to induce the formation of RR. In contrast to RR, the production of retinal-loukoumasomes was unaffected. Coimmunostaining of Y79 cells for beta-III tubulin and IMPDH indicate that these cells, when treated with ribavirin, can contain both retinal-loukoumasomes and RR and that these structures are antigenically distinct. Subcellular fractionation studies indicate that ribavirin increased the RR subcomponent, IMPDH, in the nuclear fraction of Y79 cells from 21.3 ± 5.8% (0 mM ribavirin) to 122.8 ± 7.9% (1 mM ribavirin) while the subcellular localization of the retinal-loukoumasome subcomponent tubulin went unaltered. Further characterization of retinal-loukoumasomes in retinoblastoma cells reveals that they are intimately associated with lamin folds within the nuclear envelope. Using immunofluorescence and the in situ PLA in this cell type, we have observed colocalization of beta-III tubulin with MAP2. As MAP2 is a microtubule-associated protein implicated in microtubule crosslinking, this supports a role for microtubule crosslinkers in the formation of retinal-loukoumasomes. Together, these results suggest that loukoumasomes and RR are distinct subcellular macromolecular structures, formed by different cellular processes and that there are other loukoumasome-like structures within retinal tissues and cells.

## Introduction

Rods and rings (RR), first described in 2005 [1], are subcellular structures that occur as toroidal bodies and rods (for recent reviews see [2, 3]). RR have been observed in a variety of species and cell types including human cancer cell lines, primary rodent cardiomyocytes and embryonic stem cells, drosophila follicle cells, nurse cells and oocytes as well as yeast and bacteria cells. RR are present in two filamentous forms: linear “rod” structures or toroidal “ring” structures, that range in size from less than 1 μm to 50 μm in size [2–4]. Structures, thought to be intermediate between rods and rings, with a “sewing pin” and “figure eight” shape, have also been reported [2]. These structures are found predominantly in the cytoplasm, although smaller versions have been found in the nucleus [5] (for review see [3]). The enzyme inosine monophosphate dehydrogenase (IMPDH) is a major component of RR [1, 4, 6–8] and functions primarily in the GTP biosynthesis pathway by catalyzing the rate-limiting conversion of inosine monophosphate into xanthosine monophosphate. Two isotypes of IMPDH, IMPDH1 and IMPDH2, are ubiquitously expressed in mammals, with 84% amino acid identities [9–12]. While IMPDH2 is predominantly expressed in most neoplastic and proliferating differentiated cells [9, 10, 13, 14], IMPDH1 is expressed around ten times higher than IMPDH2 in differentiated retinal cells [15]. Interestingly, mutations in the IMPDH1 gene, but not IMPDH2, result in the occurrence of a number of closely related retinal diseases [16–19]. Studies on RR have used cell cultures in which IMPDH2 is the higher expressed isotype [3–5, 8, 20]. However, IMPDH1 has also been shown to form RR structures [21, 22]. The IMPDH inhibitor, ribavirin, has been shown to induce the formation of RR as have cytidine triphosphate synthetase (CTPS) inhibitors, and glutamine deprivation [4]. Autoantibodies to IMPDH in RR have been found in hepatitis-C positive patients who were taking ribavirin and interferon-alpha; the formation of RR appears to be independent of infection with the hepatitis C virus [6, 20, 23]. Both ribavirin and interferon-alpha were subsequently tested in vitro to determine their effect on cultured cells; the IMPDH inhibitor ribavirin induced formation of RR in >95% of cultured cells while interferon-alpha had no effect [4, 6].

Less work has been performed on similar cytoplasmic structures called loukoumasomes, a name that originated from the Greek loukoumas (doughnut) and soma (body) [24]. Loukoumasomes are found in a subset of adrenergic neurons within rat sympathetic ganglia. They exist in a perinuclear location, adjacent to the Golgi apparatus and in cell periphery up until the axon initial segment [24]. Loukoumasomes are generally toroidal in the perinuclear location and linear in the cell periphery, but they can also have variant shapes thought to be intermediate between rods and toroids [24]. Toroidal loukoumasomes are predominant and have a diameter of around 6 μm [24]. To date, only one beta-III tubulin antibody (SDL.3D10) has been able to detect loukoumasomes. In rat neuronal sections, loukoumasomes did not bind TUJ1, another widely used beta-III tubulin antibody raised against a nearly identical C-terminal isotype-defining epitope. This raised doubt as to whether beta-III tubulin was indeed a true component of loukoumasomes [24]. Other components of loukoumasomes include gamma-tubulin, myosin IIb and cenexin. Due to their different cellular locations and their composition, loukoumasomes are thought to be dynamic structures, moving throughout the cell [24]. Although the function of the loukoumasome is unclear, it is believed to be an intracellular transport structure [24].

**Fig 1:**
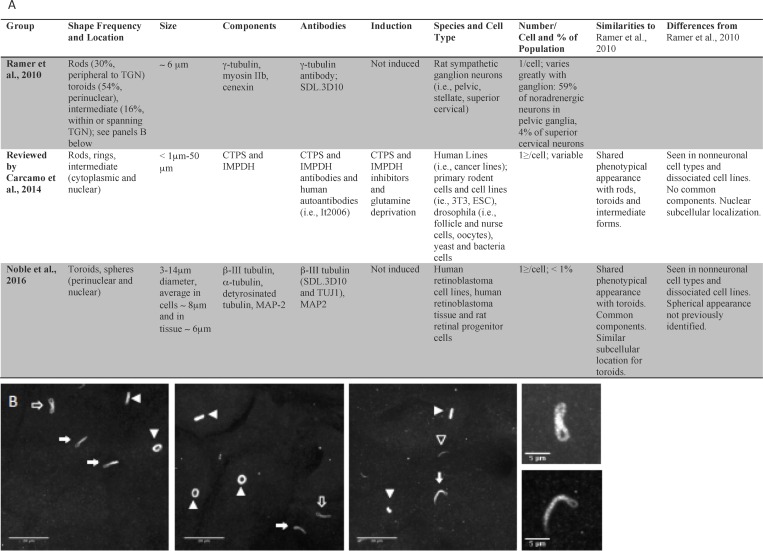
A Comparison table of RR with loukoumasomes. B. Morphological variants of loukoumasomes found in pelvic ganglia. Pericentrin immunolabeling followed by confocal analysis (z-stacks, maximum projection) reveal rods indicated by open white arrowhead), toroids (indicated by solid white arrowhead) and intermediate forms. Some toroids in the first three panels are “side view” images and therefore lack the stereotypical “donut” shape. Intermediate forms (described as twisted/pinched tori) include loukoumasomes that are similar to intermediate forms of RR including “sewing-pin” (solid white arrows) and “figure eight” (open white arrows) structures. A representative “figure eight” (upper) and “sewing-pin” (lower) intermediate form can be seen in the higher magnification panels on the far right. Calibration bar represents 20 μm in low magnification views and 5 μm in insets.

Initially the loukoumasome was proposed to be the same structure as the independently discovered RR. This was due to: 1) their similarity in size and shape and 2) the fact that the loukoumasome’s subcomponents were not fully characterized [2, 3, 23, 24]. However important differences have since emerged. For example, their antigenicity, their subcellular location and the cell types in which they are found all appear to be different for the loukoumasomes and RR (see Fig 1 for details). The purpose of this study is to investigate properties of RR and loukoumasomes, and determine whether they are the same or different structures. Our studies were performed in rat sympathetic neuronal tissue, human retinoblastoma tissue, human retinoblastoma cells and rat retinal progenitor cells. In retinoblastoma tissue and dissociated cells we observe tubulin-derived, toroidal, perinuclear macromolecular structures of similar size and antigenicity as those previously discovered in rat sympathetic neuronal sections. Moreover, we show that loukoumasomes in these cells and sympathetic neurons are antigenically distinct from RR. Finally, we show that they respond differently to ribavirin, a drug known to induce the formation of RR.

## Results

### Morphological Variants of Loukoumasomes are Composed of Stabilized Microtubules

**Fig 2:**
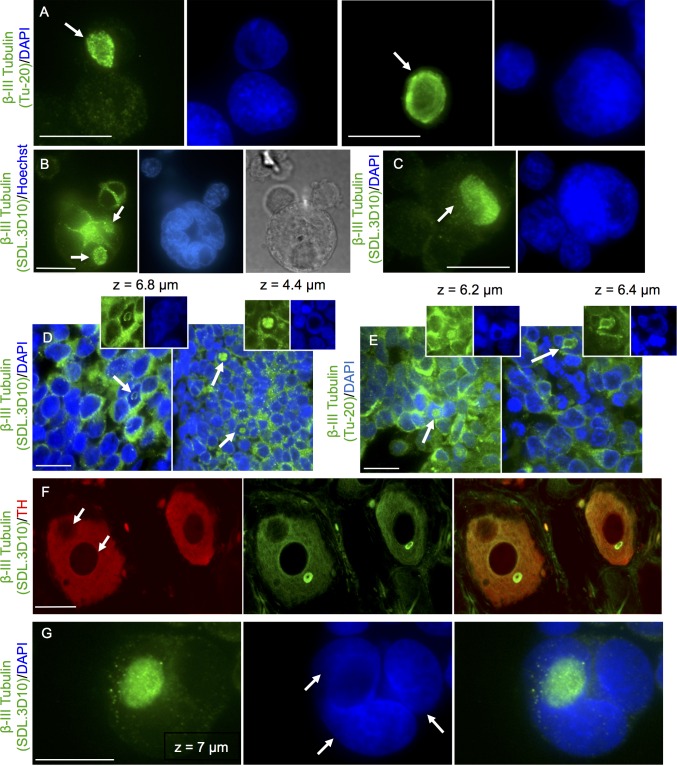
Morphological variants of loukoumasomes in retinoblastoma tissue, cell lines and sympathetic neurons as detected using two beta-III tubulin antibodies. A-C. Y79 cells were immunostained with one of two beta-III tubulin antibodies (Tu-20, A, or SDL.3D10, B-C). Nuclei were counterstained with DAPI or Hoechst (Blue). Large, toroidal shaped loukoumasomes are recognized by both antibodies (see white arrows) and are often observed within DNA impoverished pockets of the nucleus that were dark under DIC (B). D-E. Loukoumasomes though rare were observed throughout the retinoblastoma tissue samples (see white arrows highlighting loukoumasomes). Loukoumasomes were immunolabeled with an anti-beta-III tubulin antibody SDL.3D10 (D) or Tu-20 (E); nuclei were counter stained for DAPI. Loukoumasomes were observed in both Rb-positive (D, E, right panel) and Rb-negative tissue (D, E, left panel). Insets represent a single slice of the stack data illustrating the fact that large loukoumasomes are typically within a DNA impoverished (DAPI negative) region. F. Loukoumasomes appear in both bi-nucleated and mono-nucleated rat sympathetic neurons. Arrows show two nuclei in a single adrenergic neuron immunolabeled with a beta-III tubulin and tyrosine hydroxylase (TH) antibody. G. Loukoumasomes also appear in multinucleated retinoblastoma cells. A single slice of the stack data from a multinucleated cell is shown following beta-III tubulin immunolabeling and DAPI staining. Calibration bars represent 20 μm.

**Fig 3:**
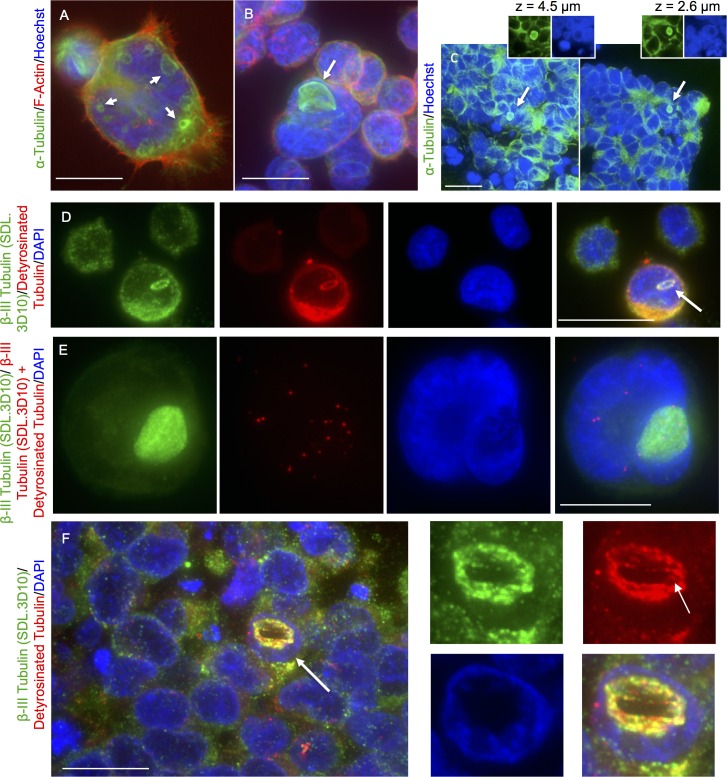
Loukoumasomes in retinoblastoma tissue and cell lines are composed of stabilized microtubules. A-B. Y79 cells were immunolabeled with an alpha-tubulin antibody and costained with phallodin and DAPI. Alpha-tubulin recognized both larger retinal-loukoumasomes (B) and smaller, more numerous retinal-loukoumasomes near the plasma membrane (see white arrows, A). C. Sections of well-differentiated human retinoblastoma tissue were immunostained for alpha-tubulin; nuclei were counterstained for DAPI. Like loukoumasomes detected with a beta-III tubulin antibody, retinal-loukoumasomes detected with an alpha-tubulin antibody were observed throughout the tissue (see white arrows highlighting loukoumasomes). D-F. Y79 cells and retinoblastoma tissue were immunolabeled with beta-III tubulin (SDL.3D10) (D-F) and detyrosinated tubulin (D,F), and costained with DAPI. Calibration bars represent 20 μm. E. Representative images showing the interaction between beta-III tubulin and detyrosinated tubulin in Y79 cells as revealed by the in situ PLA together with beta-III tubulin immunofluorescence. The strong reaction product in the PLA indicates that the two proteins are in close proximity (i.e., interacting). The fact that some red fluorescent dots occur where loukoumasomes are visualized indicates that both proteins are in close proximity at loukoumasomes. Negative PLA controls obtained by omitting one of the two primary antibodies are found in the supplementary data (see S1 Fig).

Loukoumasome numbers, reported by Ramer et al., 2010, varied in the different rat sympathetic tissue that was examined, ranging from 59% of the adrenergic neurons in the pelvic ganglion to 4% of the adrenergic neurons within the superior cervical ganglion (see Fig 1) [24]. Retinoblastoma loukoumasomes (retinal-loukoumasomes) appeared infrequently in Y79 retinoblastoma cells (less than one percent of the total cell population) and retinoblastoma tissue samples (Figs 2 and 3). The toroids we found within retinoblastoma tissue and cells were similar in size and shape to the toroids found in rat sympathetic neurons. However, in retinoblastoma cells, the toroids were more varied in size. The average diameter in dissociated retinoblastoma cells (Y79 cells), ranged from 3.24–13.74 μm with an average size of 7.63±0.42 SEM (n = 51 from 3 or more cell platings), while the average diameter in tissue ranged from 3.09–13.56 μm with an average diameter of 5.61±0.31 SEM (n = 43 cells from 3 or more tissue slices; see Fig 1 for summary). Moreover, morphological variants of retinal-loukomasomes were observed. Retinal-loukoumasomes usually appeared as regular and irregular toroids or spheres with staining patterns that were punctate in some cases while being tubular and continuous in others (Figs 2 and 3). With the exception of a single cell, we did not observe loukoumasomes in R28 rat retinal precursor cells. Although most retinal-loukoumasome-containing cells had only one loukoumasome, some were found to have two or more (Figs 2 and 3). Neuronal-loukoumasomes had been previously detected with only one beta-III tubulin antibody (SDL.3D10) making it impossible to conclude that beta-III tubulin or other related microtubule associated tubulins were indeed a subcomponent. We used both the beta-III tubulin antibodies SDL.3D10 and Tu-20 (similar to TUJ1) as well as an alpha-tubulin antibody to detect loukoumasomes in retinoblastoma cell lines (Figs 2 and 3). In general, retinal-loukoumasomes detected with the beta-III tubulin antibody SDL.3D10, were more granular, while those detected with an alpha-tubulin antibody appeared more filamentous. Additionally, all retinal-loukoumasomes had a circular morphology and while some were clearly toroidal, others were more spherical (Figs 2 and 3). The morphological distribution in cultured Y79 cells was 56% toroids and 44% spheres (n = 61 from 3 or more cell platings) while in retinoblastoma tissue it was 31% toroids and 69% spheres (n = 61 from 3 or more tissue slices). Unlike rat sympathetic tissue (Fig 1), we rarely observed loukoumasomes that appeared rod-like or as twisted/pinched tori in retinoblastoma cells (we found only one loukoumasome having a figure eight conformation). Loukoumasomes were also present in retinoblastoma tissue samples that expressed the retinoblastoma tumour suppressor protein (Rb) and samples that did not (Fig 2D and 2E, samples that are Rb-positive are shown in the right panels while Rb-negative samples are shown in the left panels). Loukoumasomes were immunolabeled with an anti-beta III tubulin antibody SDL.3D10 (Fig 2D) or Tu-20 (Fig 2E). In retinoblastoma cells, we have also found that loukoumasomes are present in both mononucleated and multinucleated cells which is consistent with the findings in rat sympathetic tissue (Fig 2F and 2G, arrows indicate two to three nuclei in a single neuron) [24]. Twenty percent of the cultured Y79 cells that contained loukoumasomes were multinucleated (n = 61 cells from 3 or more cell platings). Given that only 2% of the overall population of Y79 cells are multinucleated, it is significant that a relatively high percentage of retinal-loukoumasome-bearing cells were multinucleated.

Y79 cells immunolabeled with an alpha-tubulin antibody recognized both larger retinal-loukoumasomes (Fig 3B) and smaller, more numerous retinal-loukoumasomes near the plasma membrane (see white arrows, Fig 3A). Sections of well-differentiated human retinoblastoma tissue were also immunostained for alpha-tubulin. Like loukoumasomes detected with a beta-III-tubulin antibody, retinal-loukoumasomes detected with an alpha-tubulin antibody were observed throughout the tissue (see white arrows highlighting loukoumasomes). In both Y79 cells and retinoblastoma tissue, beta-III tubulin colocalized with detyrosinated tubulin in loukoumasomes (Fig 3D–3F). Detyrosination occurs when the C-terminal tyrosine of an alpha-tubulin in microtubule polymers is removed [25]. Colocalization was either determined using immunofluorescence alone (Fig 3D and 3F) or together with PLA (Fig 3E). The PLA technique uses a pair of oligonucleotide labeled antibodies that bind in close proximity (30–40 nm apart) to two different proteins. Colocalization of detyrosinated tubulin and beta-III tubulin using both immunofluorescence and PLA is consistent with alpha-tubulin staining of retinal-loukoumasomes and loukoumasomes being microtubule derived. As expected, the PLA indicates that these proteins colocalize in areas of the cell other than loukoumasomes. This is likely owing to the complex distribution of these cytoskeletal proteins to many locations within the cell.

### Loukoumasomes and RR are Antigenically Distinct

**Fig 4:**
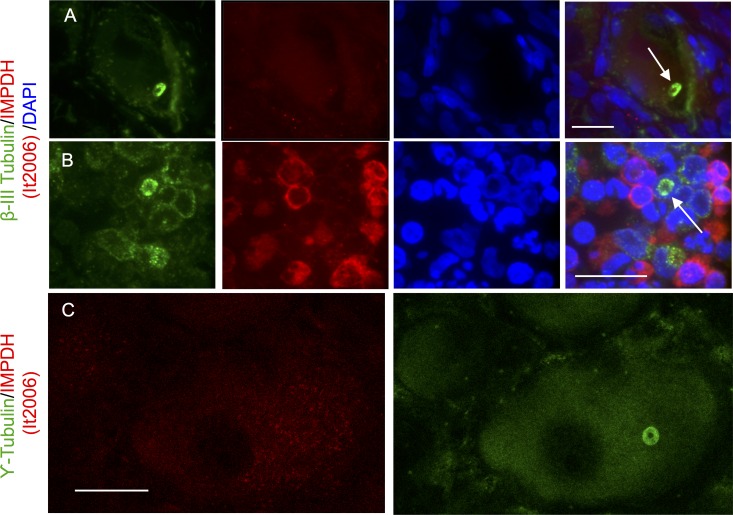
Loukoumasomes and RR are Antigenically Distinct A. Adult rat sympathetic ganglia were co-labelled with the beta-III tubulin antibody, (SDL.3D10) known to recognize loukoumasomes and the anti-RR/IMPDH antibody, (It2006) and stained with DAPI. A single large perinuclear toroidal loukoumasome can be seen within a large sympathetic neuronal cell soma. This neuronal cell is surrounded by glia (as evident by small intensely stained nuclei). Although the loukoumasome stained intensely with the beta-III tubulin antibody it was not immunolabeled with the anti-IMPDH antibody. B. Retinoblastoma tissue was also immunolabeled with a beta-III tubulin antibody (SDL.3D10) together with the anti- IMPDH antibody (It2006). Arrows depict a loukoumasome that is immunolabeled with an anti-loukoumasome antibody (SDL.3D10) but not the anti-RR antibody (It2006). RR immunostaining was not expected in untreated retinoblastoma tissue. However, intense cytosolic immunoreactivity with the anti-IMPDH antibody was observed in cells having condensed nuclei. This cytosolic staining can be seen encircling the nucleus but does not immunolabel loukoumasomes. C. Adult rat sympathetic ganglia were co-labeled with the gamma-tubulin antibody (also known to recognize loukoumasomes) and the anti-RR antibody (It2006) and stained with DAPI. Loukoumasomes though positive for gamma-tubulin, did not contain IMPDH.

Loukoumasomes, first discovered in differentiated adult rat sympathetic neurons [24], were immunolabeled with the anti-beta-III tubulin antibody SDL.3D10. If loukoumasomes and RR are the same structure, then the anti-RR human serum (It2006) should recognize loukoumasomes, and the anti-loukoumasome beta-III tubulin antibody should recognize RR. Therefore, we coimmunolabeled rat sympathetic neuronal sections with both antibodies. Shown is a flattened confocal stack of a single, toroidal loukoumasome, present within a large sympathetic neuron (Fig 4A). Although intensely stained with the anti-beta-III tubulin antibody SDL.3D10, it is not immunoreactive with the IMPDH antibody (Fig 4A). Likewise, retinoblastoma tissue was also immunolabeled with these same antibodies and the macromolecular structures that resemble loukoumasomes were only immunolabeled with the beta-III tubulin antibody (Fig 4B); the anti-IMPDH antibody It2006 gave diffuse cytoplasmic staining. Similarly, in rat sympathetic neurons, loukoumasomes, though clearly labelled with gamma-tubulin, were not immunolabeled with the anti-IMPDH antibody It2006 (Fig 4C). Together, these results indicate that although tubulin positive neuronal-loukoumasomes and retinal-loukoumasomes were clearly present in both neuronal and retinoblastoma tissue, respectively, they did not exhibit anti-RR immunoreactivity. These results indicate that RR are antigenically distinct from the two loukoumasome types, being specifically recognized only by their respective antibodies.

### Ribavirin Induces RR but not Loukoumasome Formation in Retinal Cell Lines

**Fig 5:**
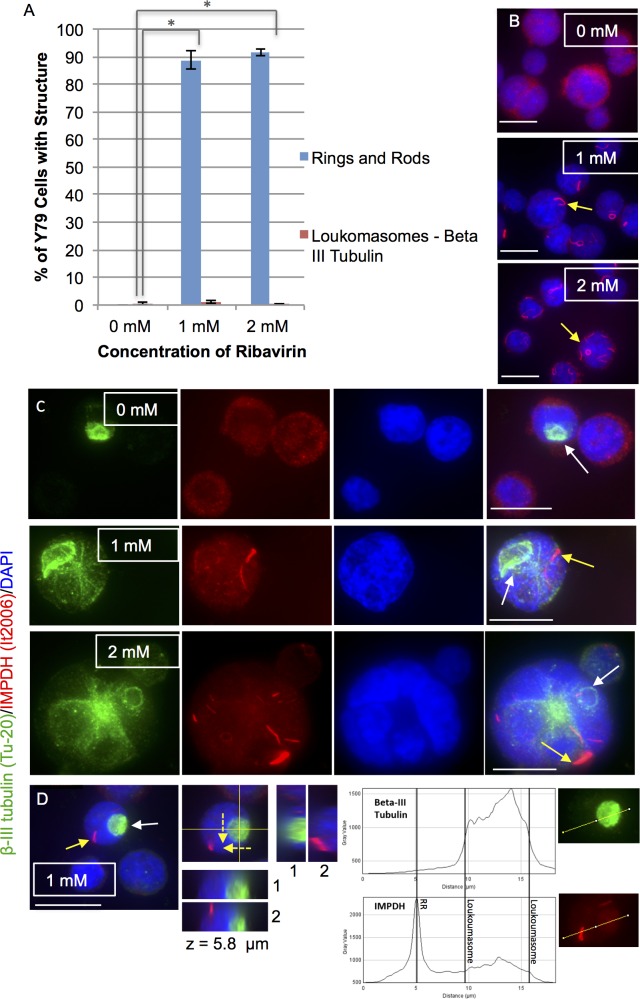
Induction of RR, but not loukoumasomes, in retinoblastoma cells with ribavirin. A. A 24 hour exposure to ribavirin (an IMPDH inhibitor) increased the number of Y79 cells with RR but not retinal-loukoumasomes. Loukoumasomes were quantitated using an anti-beta-III tubulin antibody (clone Tu-20) and RR were quantitated using an anti-IMPDH antibody (anti-RR serum It2006). Data are represented as the mean ± SEM from 4 independent experiments with greater than 400 cells counted per treatment group. * p < 0.01, different from untreated control. Significance was determined using an ANOVA and Fisher’s (LSD) test. B. Y79 cells were immunolabeled with an IMPDH autoantibody It2006 (red) and counterstained with DAPI (blue) following exposure to ribavirin (1 mM and 2 mM) or control (0 mM) for 24 hours. Yellow arrows depict an example of RR in the 1 mM and 2 mM treatment groups. No RR were present in the control group. C. Representative figures of Y79 cells, immunolabeled with a monoclonal antibody against beta-III tubulin (clone TU-20; green) together with an IMPDH autoantibody It2006 (red). Nuclei were counterstained with DAPI (blue). This was done following exposure to 1 mM and 2 mM ribavirin for 24 hours or vehicle control. White arrows highlight loukoumasomes, while yellow arrows indicate examples of RR. D. A confocal stack of a Y79 cell (left panel) along with orthogonal views of the same cell (right panel) at a given z position (z = μm). Each successive orthogonal step (labelled 1–2) is 7.36 μm (for the yz orthogonal views) or 2.56 μm (for the xz orthogonal views) apart; broken arrows indicate the direction in which steps were taken. The orthogonal views indicate that the loukoumasome and RR are distinct structures in separate sections of the cell. Tubulin and IMPDH It2006 intensity profiles on this same cell were performed. Fluorescence intensities indicate that the loukoumasome (green fluorescence) and the RR (red fluorescence) are distinct structures. Calibration bars represent 20 μm.

Earlier studies have shown that CTPSI inhibitors as well as the IMPDH inhibitor ribavirin, induced RR assembly in greater than 95% of cancer cell lines tested [4]. If indeed loukoumasomes and RR were related or overlapping cytoplasmic filamentous structures, both would be expected to respond similarly to these enzyme inhibitors. Retinal-loukoumasomes were quantitated using an anti-beta-III tubulin antibody (clone Tu-20) and RR were quantitated using an anti-IMPDH antibody (anti-RR serum It2006). A twenty-four hour exposure to ribavirin increased the number of RR but did not change retinal-loukoumasome numbers within Y79 cells. Specifically, in control Y79 retinoblastoma cells, RR were absent; when 1 and 2 mM ribavirin was added, the amount of cells containing RR increased to approximately 89 and 92 percent, respectively (Fig 5A). The induction of RR and the fact that there was not a significant difference between 1 and 2 mM ribavirin concentrations was similar to that observed in other human cancer cell lines tested [4]. Representative figures of Y79 cells immunolabeled for IMPDH (using the anti-IMPDH autoantibody It2006; red) alone or together with beta-III tubulin (using the Tu-20 antibody; green) and counterstained with DAPI (blue) are shown. These results indicate that ribavirin increases RR formation but does not alter retinal-loukoumasomes (see Fig 5B and 5C). Sometimes, both loukoumasomes and RR were observed within the same retinoblastoma cell and are recognized selectively by either an anti-loukoumasome (SDL.3D10) or anti-RR antibody It2006 (Fig 5C and 5D). This is further evidence to suggest that loukoumasomes and RR are distinct macromolecular structures, being recognized by their respective antibodies within the same cell. A representative confocal stack of an imaged Y79 along with orthogonal views of the same cell indicates that the loukoumasome and RR are distinct structures within the cell (Fig 5D). The fact that the retinal-loukoumasomes are recognized by more than one beta-III tubulin antibody indicates that beta-III tubulin is indeed the responsible marker antigen.

### The Localization of RR but not Retinal-Loukoumasome Subcomponents Differs Following Ribavirin Treatment

**Fig 6:**
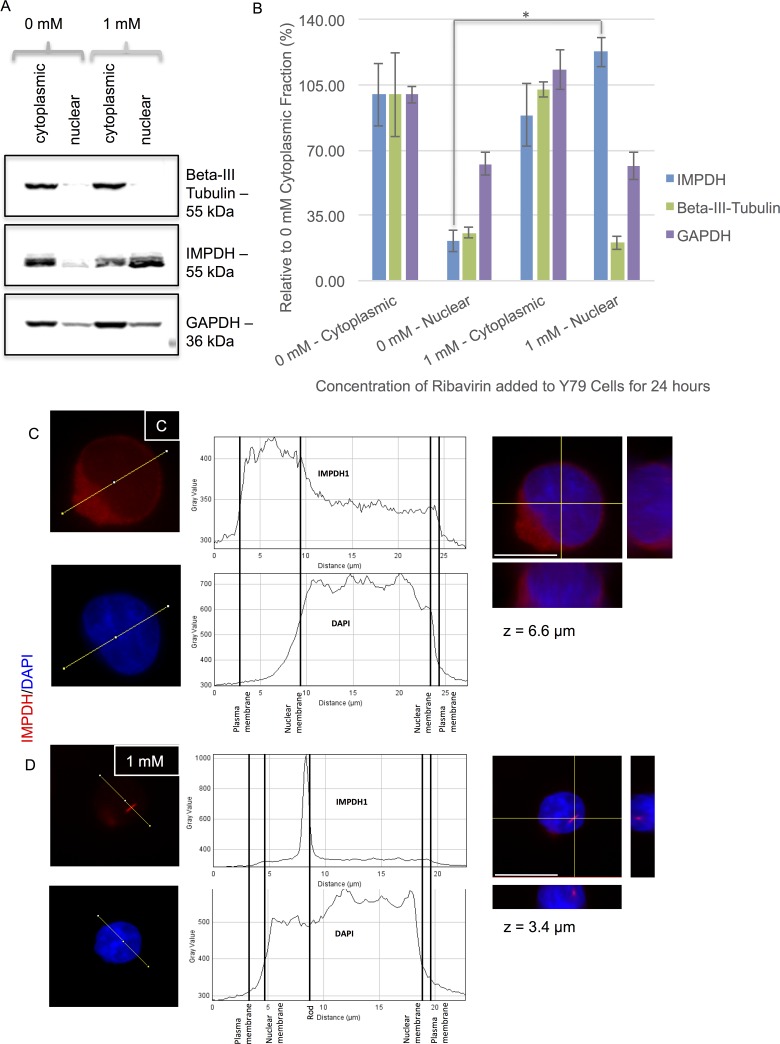
The localization of RR but not retinal-loukoumasome subcomponents differs following ribavirin treatment. A. Western blots demonstrate a marked increase in nuclear IMPDH1 in Y79 cells treated with 1 mM ribavirin for 24 hours. In contrast, ribavirin treatment did not affect beta-tubulin III or GAPDH subcellular localization. B. Densitometric analysis was performed on Western blots run in parallel and then probed with either beta-III tubulin, IMPDH1 or GAPDH. Protein expression was averaged and normalized to the untreated cytoplasmic fraction within each group. Data are represented as the mean ± SEM from 3 independent experiments. * p < 0.05, different from treated fraction in the same group. Significance was determined using an ANOVA and Fisher’s (LSD) test. C. Y79 cells were untreated (0 mM) or treated with 1 mM ribavirin. Cells were then immunostained for IMPDH and costained for DAPI to visualize RR. D. Orthogonal views of the same Y79 cells at a given z position are shown. While RR were not evident in controls, fluorescence intensities indicate that RR (IMPDH immunostaining) occur within the nucleus (DAPI positive) of some cells following ribavirin treatment.

In addition to cytoplasmic RR structures, RR have also been detected within the nucleus following treatment with CTPS or IMPDH inhibitors or by glutamine deprivation. These were somewhat smaller in size than their cytoplasmic counterpart [3]. Subcellular fractionation studies were undertaken to determine how the localization of components that make up RR (i.e., IMPDH1 or IMPDH2) or loukoumasomes (i.e., beta-III tubulin) were affected by ribarivin treatment for 24 hours (Fig 6). Western blots demonstrate a marked increase in nuclear IMPDH in Y79 cells treated with 1 mM ribavirin (Fig 6A). Densitometric analysis performed on Western blots indicates IMPDH increased from 21.3 ± 5.8% (0 mM ribavirin) to 122.8 ± 7.9% (1 mM ribavirin) in the nuclear fraction. In contrast, ribavirin treatment did not affect beta-III tubulin or GAPDH subcellular localization. No change in either beta-III tubulin or GAPDH was observed, thus confirming that only the IMPDH subcellular localization was altered by ribavirin treatment (Fig 6B). The significant increase in IMPDH in the nuclear fraction is consistent with the formation of a subcellular structure that is increased following ribavirin exposure. These results are consistent with the hypothesis that beta-tubulin III and IMPDH subcellular macrostructures are distinct. Evidence to support the nuclear localization of IMPDH after ribavirin treatment is shown in Fig 6C. The plotted fluorescence intensity indicates that the IMPDH signal formed a clear rod-shaped RR in the nucleus (Fig 6C).

### Retinoblastoma Cells Contain Perinuclear Retinal-Loukoumasomes that are Closely Associated with Lamin and Colocalize with MAP2

**Fig 7:**
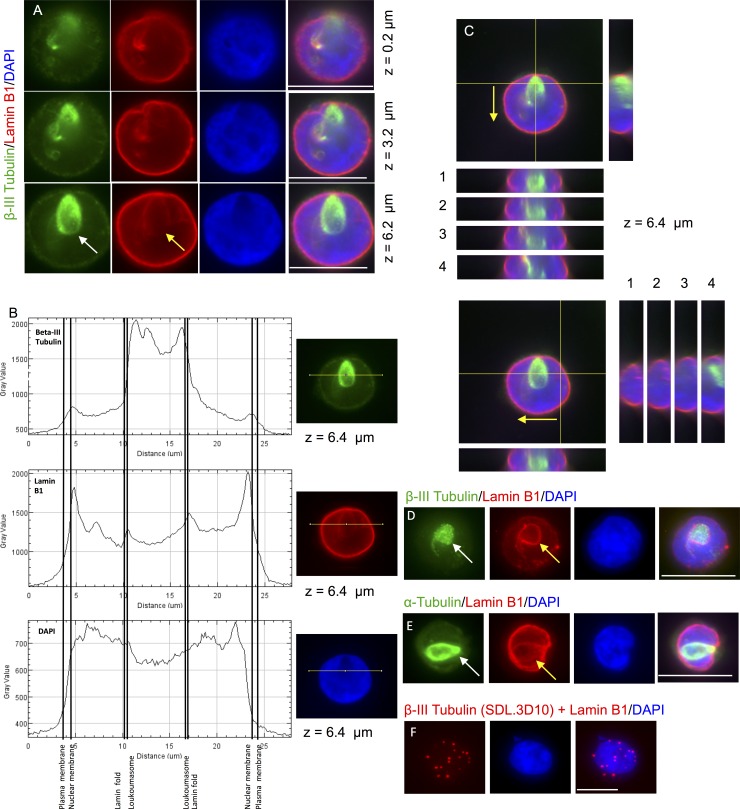
Retinal-loukoumasomes reside in the perinuclear space in close association with lamin in retinoblastoma cells. A-C. The perimeter of retinal-loukoumasomes are outlined by intense lamin B1 staining suggesting that they are perinuclear. A. Y79 cells immunostained for beta-III tubulin using the SDL.3D10 antibody (green), lamin B1 (red) and costained for DAPI (blue). The z positions of single slices from the z-stack data are indicated. White arrows highlight retinal-loukoumasomes, while yellow arrows highlight intense lamin B1 staining. B. Tubulin, lamin B1 and DAPI intensity profiles on this same cell was performed. Fluorescence intensities indicate that tubulin nestles within a lamin fold near an area of the nucleus lacking DNA labelling. C. Confocal orthogonal views of the same Y79 cell at a given z position (z = 6.4 μm) are shown. Each successive orthogonal step (labelled 1–4) is 1.6 μm apart (arrows indicate the direction in which steps were taken). An intimate association with lamin B1 folds and the loukoumasome is evident. Polymerized tubulin “tubulin tail” appears to be continuous with the toroidal loukoumasome. D. A large loukoumasome is shown in a Weri-Rb 27 cell immunolabeled for beta-III tubulin together with lamin B1 and stained with DAPI. As seen for the loukoumasome in A-C, a beta-III tubulin “tail” is also evident here. E. The lamin foldings around loukoumasomes were also evident in Y79 cells coimmunolabeled for alpha-tubulin with lamin B1 and counterstained with DAPI. F. Representative images showing the interaction between beta-III tubulin and lamin B1 as revealed by the in situ PLA. Punctate red fluorescence indicates beta-III tubulin and lamin are in close proximity.

Since most toroidal neuronal-loukoumasomes described by Ramer et al., 2010 were perinuclear, we immunolabeled beta-III tubulin together with lamin B1 to characterize association of retinal-loukoumasomes with the nuclear envelope. As Y79 cells consist mostly of a nucleus surrounded by a thin layer of cytoplasm then it would be expected to find loukoumasomes nestled within the nuclear envelope. Indeed the perimeter of retinal-loukoumasomes are outlined by intense lamin B1 staining suggesting that they are intimately associated with the nuclear envelope residing possibly on the nucleoplasmic surface of the nuclear envelope (Fig 7A). A plot of the fluorescence intensities for tubulin, lamin B1 and DAPI indicate that tubulin nestles within a lamin fold near an area of the nucleus lacking DNA labelling (Fig 7B). This is also evident by successive confocal orthogonal views showing tubulin and lamin B1 immunoreactivity, taken at z-step 6.4 μm (Fig 7C). Likewise, in another retinoblastoma line, Weri-Rb 27 cells, retinal-loukoumasomes were also intimately associated with lamin B1 (Fig 7D). In addition to the beta-III tubulin antibody (SDL.3D10), the lamin foldings were shown to be present around retinal-loukoumasomes immunolabeled with an alpha-tubulin antibody in Y79 cells (Fig 7E). A close physical association between beta-III tubulin and lamin B1 was also evident using the PLA assay (Fig 7F). In both the orthogonal view of a retinal-loukoumasome visualized with a beta-III tubulin antibody (Fig 7C) and the flattened confocal stack following immunolabeling for another retinal-loukoumasome visualized with a beta-III tubulin antibody (Fig 7D), additional polymerized tubulin (“tubulin tail”) can be seen that appears continuous with a toroidal retinal-loukoumasome. Having observed this, we subsequently determined that the percentage of retinal-loukoumasomes with microtubule fiber attachments (or “tails”) were 21% (n = 61).

**Fig 8:**
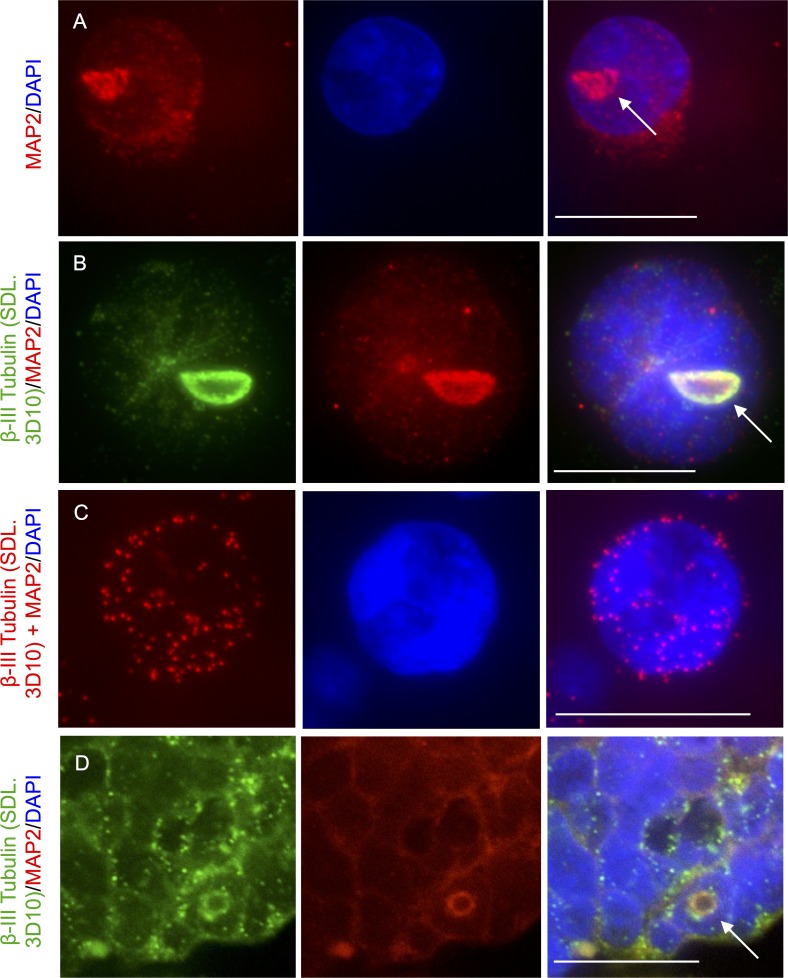
MAP2 colocalizes with loukoumasomes in retinoblastoma cells and retinoblastoma tissue. A-B. Y79 cells were immunolabeled for MAP2 alone (A) or together with beta-III tubulin using the SDL3D10 antibody (B) and costained with DAPI. C. Colocalization of beta-III tubulin and MAP2 in Y79 cells was also observed using the in situ PLA. Red fluorescent signals indicate that both beta-III tubulin and MAP2 are closely associated in Y79 cells. D. Retinoblastoma tissue was coimmunolabeled for beta-III tubulin using the SDL.3D10 antibody together with MAP2 and costained with DAPI (blue). The arrows depict retinal-loukoumasomes.

Both MAP2 and the human MAP65, known as PRC1, are proteins that crosslink microtubules [26–29] and alter microtubule flexibility [30, 31]. Kinesin motors power microtubule looping in cultured cells [32] and filament-gliding assays have shown that MAP65 cooperates with kinesin motors to form microtubule loops when microtubules slide past each other [29]. Both MAP2 and PRC1 were coimmunolabeled with beta-III tubulin (SDL.3D10) to determine if they had a possible relationship with retinal-loukoumasomes. A MAP2 antibody was used to detect these macromolecular structures in retinoblastoma cells and tissue (Fig 8A, 8B and 8D). To ensure that the MAP2 antibody signal at the loukoumasome was not due to bleed through from the beta-III tubulin antibody signal, MAP2 immunolabelling was performed alone (Fig 8A) and together beta-III tubulin (Fig 8B and 8D). As was observed for tubulin immunolabelling of retinal-loukoumasomes, MAP2 immunolabelling revealed different morphological variants that included irregular toroids and spheres (Fig 8A, 8B and 8D). Although a MAP2 antibody immunolabeled retinal-loukoumasomes, a PRC1 antibody (Santa-Cruz Biotechnology CAT# sc-8356), did not. This was true when MAP2 antibodies were used alone or together with beta-III tubulin antibodies (Fig 8). Colocalization of beta-III tubulin and MAP2 was also observed using the in situ PLA assay. As neither of these antibodies targets loukoumasomes exclusively, beta-III tubulin and MAP2 colocalization was also observed apart from the loukoumasomes.

## Discussion

Loukoumasomes and RR were previously inferred to be the same structures largely due to their shared distinct shape (“rings” or toroids, rods and intermediate shapes), their large size, and the fact that loukoumasomes subcomponents were not fully characterized. Phenotypic similarities between the two structures have been previously reviewed [3]. In the present study, we identified RR and loukoumasomes as distinct toroidal and spherical structures within human retinoblastoma tissue and dissociated human retinoblastoma cells. In retinoblastoma tissue and cells, we observed toroidal, perinuclear, macromolecular structures of similar size and antigenicity to loukoumasomes previously reported by Ramer et al., 2010 (see Fig 1). Following further immunocytochemical characterization, we found that these structures are derived from tubulins associated with microtubules and that they colocalize with MAP2, a microtubule associated protein implicated in crosslinking microtubules and potentially locking them in defined shapes. We show that RR and loukoumasomes (retinal- and neuronal-) are spatially and antigenically distinct, subcellular structures. Furthermore, we show that retinal-loukoumasomes respond differently to ribavirin, a drug that inhibits IMPDH and induces RR formation. In rat sympathetic neurons and retinoblastoma cells the anti-RR antibody It2006 did not recognize loukoumasomes. Rather, in retinoblastoma cells, retinal-loukoumasomes were immunolabeled by tubulin antibodies including the anti-neuronal-loukoumasome beta-III tubulin antibody SDL.3D10, a second beta-III tubulin antibody Tu-20, an alpha-tubulin antibody and a detyrosinated tubulin antibody. Furthermore, in Y79 cells, ribavirin treatment increased the percentage of RR-bearing cells to approximately 90% while having no effect on retinal-loukoumasome-bearing cells. In R28 cells, a rat retinal precursor cell line, ribavirin also induced a visible increase in RR but did not alter retinal-loukoumasomes (S2 Fig). Likewise, although ribavirin increased the RR subcomponent IMPDH in the nuclear fraction of Y79 cells, the subcellular localization of the loukoumasome subcomponent tubulin was unaffected.

When comparing the loukoumasomes found in retinoblastoma cells to those first reported by Ramer et al., 2010 in sympathetic neurons, there are some noteworthy similarities and differences. Within both rat neuronal tissue and human cancer lines, toroidal loukoumasomes were often located in a perinuclear location and were immunostained for beta-III tubulin. Also, in both the retinoblastoma cell lines and rat sympathetic neuronal tissue these macromolecular structures appeared in both mononucleated and multinucleated cells. Differences include their nuclear location, loukoumasome numbers in dissociated cells, the shape of loukoumasomes found in these two different cell sources and their antigenicity with other cytoskeletal associated proteins. Unlike neuronal-loukoumasomes that were only cytoplasmic, retinal-loukoumasomes appeared both perinuclear and intranuclear. The intranuclear localization of retinal-loukoumasomes was suggested by their intimate association with lamin, a protein found on the nucleoplasmic side of the nuclear envelope. Dissociated retinoblastoma cells maintained these macromolecular structures but when explants of rat neuronal tissue were dissociated, loukoumasomes disappeared [24]. In rat neuronal tissue samples, loukoumasomes appeared toroidal, rod-shaped and in intermediate forms. In retinoblastoma cells, loukoumasomes were either toroidal or spherical in shape and intermediate forms were extremely rare (see Fig 1). Also, in rat neuronal tissue, these macromolecular structures were isolated from other polymerized tubulin in the cell, while in retinoblastoma cells they sometimes appeared continuous with polymerized tubulin “tails”. Finally, although MAP2 and detyrosinated tubulin colocalized with beta-III tubulin in retinal-loukoumasomes, these proteins did not appear to immunolabel neuronal-loukoumasomes (S3 Fig). Together these differences indicate that neuronal- and retinal-loukoumasomes may indeed be distinct structures. A detailed analysis is needed to compare and contrast these two structures and to rule out whether or not technical issues (i.e., differences in species and sample preparation) underlie differences in immunolabelling that we have observed between neuronal- and retinal-loukoumasomes.

In addition to loukoumasomes, tubulin-containing inclusions have been observed in either the human brain [33] or human neoplasms [34]. These proteinaceous structures were also detected with the same human beta-III tubulin antibody (SDL.3D10) used to detect loukoumasomes. In the human brain, these inclusions were found in neurons and ependymal cells [33]. Inclusion-bearing neurons were widespread having a heterogeneous topographic pattern of distribution [33]. Although immunostaining of these structures was observed in the nuclei and in the perikarya, investigators have focused on intranuclear immunostaining referring to this as “intranuclear inclusions” [33]. Tubulin immunoreactive intranuclear inclusions have also been found to exist in human intracranial neoplasms such as ependymomas and gangliogliomas [34]. Intranuclear inclusions in the human brain cells and their neoplastic counterparts were varied in shape and included rod shaped, polygonal or irregular structures [24, 33, 34]. They were also found to range in length from 1.0–9.0 μm and in width from 0.1 to 0.6 μm [33]. Indeed these inclusions have been likened to intranuclear inclusions called “intranuclear rodlets,” that have been described at the light microscopic level [35] and subsequently characterized using electron microscopy [36–38]. These intranuclear inclusions have been shown to be partially composed of microtubules [39]. Although our studies clearly show that RR and loukoumasomes are distinct macromolecular structures, future studies are required to determine the relationship between loukoumasomes, tubulin immunoreactive inclusions and other intranuclear inclusions found in these independent laboratories.

Dynamic movement of microtubules may underlie both the formation and function of tubulin derived loukoumasomes. In the past, although microtubules have been thought of as stiff structures, they have been shown to buckle and loop in cultured cells [40–43]. Our finding that retinal-loukomasomes colocalize with MAP2 may be significant as MAPs may alter the ability of microtubules to form loops in at least a couple of different ways. First, MAPs stabilize microtubule loops through crosslinking and second, MAPs alter the flexibility of the microtubules themselves. For example, MAP2 has been shown to crosslink dendritic microtubules [26, 27] while the human MAP65, PRC1, has been shown to crosslink antiparallel microtubules that have looped back on themselves [28, 29]. Furthermore, the MAPs tau and MAP2 stiffen microtubules [30] while members of the conserved MAP65 family increase flexibility [31].

Microtubule looping and buckling is thought to by powered by motor proteins “piggybacking” cargo along moving microtubules [43]. This would be consistent with an earlier hypothesis that loukoumasomes may act as an intracellular transport machine in neuronal cells [24]. Mechanisms giving rise to loukoumasomes may also be shared by other intranuclear [39] inclusions shown to contain tubulin or microtubules [33, 34, 39]. Whether or not some of these dynamic processes underlie the formation and function of loukoumasomes and other intranuclear inclusions is yet to be determined.

## Methods

### Cell Culture and Drug Exposure

We used established retinoblastoma cell lines [44–46] and immortalized, rat, retinal, precursor cells [47]. Human retinoblastoma cell lines (Y79 and Weri-Rb27) and R28 cells were cultured using DMEM+Glutamax (GIBCO), with 10% fetal bovine serum and gentamicin (50 μg/ml) as described. [48, 49] R28 cells were passaged onto poly-D-lysine coated coverslips in 6-well plates prior to ribavirin exposure at a density of 6.0×10^5^ cells per well. Cells were incubated at 37°C and 5% CO_2_. High-density retinoblastoma cultures were split 1:2 every one to two days while low-density cultures were split 1:10 once a week as previously described [48]. Ribavirin (Sigma-Aldrich; R9644) was solubilized in DMEM+Glutamax (GIBCO) and added to cells for 24 hours at a final concentration of 1 and 2 mM.

### Immunohistochemistry

Prior to undertaking these studies, ethical approval was obtained from the University of British Columbia Ethical Review Board and from the Trinity Western University Ethical Review Board for the use of human paraffin embedded tissue. Written informed consent from the donor or the next of kin was obtained for the use of this sample in research. Paraffin embedded sections were deparaffinized and subjected to a citrate buffer (pH 6.0) antigen retrieval step as previously described [50]. Hydrated sections were then blocked in 5% normal goat serum in 0.1% BSA/TBS-Tween and solubilized in 0.2% Triton X-100 in PBS. Sections were incubated in PBS with 1% normal goat serum in 0.1% BSA/TBS-Tween containing a primary antibody to MAP2 (Abcam CAT# ab32454) or beta-III tubulin (Sigma-Aldrich Cat# T8660) overnight at 4°C and incubated in Alexa fluor® secondary antibodies in PBS for one hour at 37°C. The samples were then mounted with Vectashield Mounting Medium with DAPI (Vector Laboratories Cat# H-1200), and covered with coverslips.

The University of British Columbia Animal Care Committee specifically approved these studies involving rodents, prior to the start of all experiments. Pelvic ganglia were dissected from adult male Wistar rats that were transcardially perfused with phosphate buffered saline followed by 4% paraformaldehyde (PFA) in 0.1 M phosphate buffer (PB). The dissected tissue was post-fixed overnight then cryprotected in 20% sucrose in 0.1 M PB at 4°C for a minimum of 24 hours before being flash frozen in Cryomatrix (Thermo Scientific Cat#6769006). Sixteen micrometer tissue sections were cut, mounted and then blocked with 10% normal donkey serum in 0.2% Triton/PBS/0.1% sodium azide for 1 hour at room temperature. Sections were incubated overnight with the appropriate primary antibody, beta-III tubulin (Sigma-Aldrich Cat#T8660), gamma tubulin (Abcam Cat#ab11317), pericentrin 1 (Santa Cruz, E-17 sc-28142 lot#H2806), tyrosine hydroxylase (Millipore Cat#AB152) or anti-RR antibody (It2006), diluted in 0.2% Triton/PBS/0.1% sodium azide and subsequently incubated for 2 hours with Cy^TM^3 and Alexa Fluor® 488 secondaries. The sections were coverslipped using Shandon Immu-mount (Thermo Scientific Cat#9990402) and imaged on a Zeiss Axio Observer Z1 inverted spinning disk confocal microscope.

### Immunocytochemistry

Retinoblastoma cells grown in suspension were plated onto poly-D-lysine coated 12-well slides just prior to fixation [48]. Retinoblastoma cells and R28 cells were fixed with 4% PFA and permeabilized using 0.02% Triton/TBS as described [48]. Alternatively, cells were fixed and permeabilized with ice-cold methanol followed by acetone at 4°C. Cells were then blocked with 5% NGS in 0.1% BSA/TBS-Tween and incubated overnight at 4°C with primary antibodies in incubating solution containing 1% NGS in 0.1% BSA/PBS-Tween. The antibodies against the following were used: IMPDH1 (Proteintech Cat#22092-1-AP; an antibody raised against the entire IMPDH1 fusion protein), IMPDH2 (Abcam Cat#ab75790), alpha-tubulin (Sigma-Aldrich Cat#T6199), beta-III tubulin (Millipore Cat#MAB1637 and Sigma-Aldrich Cat#T8660), autoantibody It2006 (a kind gift from Edward K. L. Chan), lamin B1 (Abcam Cat#ab16048), detyrosinated tubulin (Millipore Cat#AB3201) and MAP-2 (Abcam Cat#ab32454). Cells were then washed with 0.1% BSA/PBS-Tween and incubated for one hour at 37°C with Alexa fluor® secondary antibodies. Cells were washed with 0.1% BSA/PBS-Tween and mounted with Vectashield Mounting Medium containing DAPI (Vector Laboratories Cat#H-1200) for nuclear staining.

### In Situ Proximal Ligation Assay

The colocalization of beta-III tubulin together with detyrosinated tubulin, MAP2 and lamin B1 was assessed using the in situ PLA (Duolink® In Situ Red Starter Kit, Sigma-Aldrich) in Y79 cells. This technology allows detection of protein-protein interaction in fixed cells using a pair of oligonucleotide-labelled secondary antibodies (PLA probes) that generate a signal only when the two PLA probes have bound to two primary antibodies that are in close proximity to one another. The signal from each pair of PLA probes is visualized as an individual fluorescent spot. Duolink® in situ PLA was performed as recommended by the manufacturer. Y79 cells were prepared as described above for immunofluorescence. Primary antibodies used were a mouse anti-beta-III tubulin antibody clone SDL.3D10 together with a rabbit anti-detyrosinated tubulin, anti-MAP2 or anti-lamin B1 antibody. When in situ PLA was done together with immunofluorescence of beta-III tubulin, the secondary proximity probes were incubated with the Alexa fluor® secondary antibody used to visualize beta-III tubulin staining. Negative controls were performed omitting one or both of the two primary antibodies (S1 Fig).

### Confocal Microscopy

Cells were viewed under an Olympus IX81 inverted microscope equipped with the Olympus DSU (Disk Scanning Unit) spinning disk confocal. Images taken using the Olympus IX81 inverted microscope, were analyzed using ImageJ and Metamorph Premier software. Loukoumasomes and RR subcellular localization were analyzed using z-stacks of fluorescent images. Unless otherwise stated, flattened z-stacks, with maximum projection are shown.

### Cell Fractionation and Western Blotting

Nuclear and cytoplasmic extracts were isolated according to the method of Schreiber et al., 1989 [51, 52]. Cells were pelleted by centrifugation, washed in TBS and resuspended in lysis buffer (10 mM HEPES pH 7.9; 10mM KCI; 0.1 mM EDTA; 0.1 mM EGTA; 1 mM DTT; 0.5 mM PMSF). The cells were incubated on ice for 15 min, after which the lysate was centrifuged. Following centrifugation, the nuclear pellet was washed and resuspended in 50 μl ice-cold buffer C (20 mM HEPES pH 7.9; 0.4 M NaCl; 1 mM EDTA; 1 mM EGTA; 1 mM DTT; 1 mM PMSF). The nuclear pellet was then incubated on ice for 15 min. followed by centrifugation. Twenty micrograms of each cell fraction protein were run on Western blots. Cytoplasmic and nuclear fractions were run in parallel and were probed with a mouse anti-beta-III tubulin antibody, rabbit anti-IMPDH1 antibody (as previously described) or rabbit anti-GAPDH antibody (1:600; Santa Cruz) as a control for total protein expression. The proteins were detected using Biorad Clarity^TM^ Western ECL substrate and visualized by Biorad ChemiDoc XRS+ with Image Lab^TM^ Software (Mississauga).

## Supporting Information

S1 Fig In Situ PLA control experiments. All antibodies used for the in situ PLA colocalization studies were also tested in single staining experiments. In Y79 cells, single primary antibodies were incubated together with both the anti-mouse and anti-rabbit PLA probes (see panels 1–4). Controls were also performed for these probes by omitting both primary antibodies in the incubations (see panel 5).
(TIF)

S2 Fig Induction of RR but not loukoumasomes in R28 cells treated with ribavirin. A-C. Representative composites of retinal R28 cells treated with ribavirin (1 mM or 2mM) or untreated control (0 mM) for 24 hours. Cells immunolabeled with either a human IMPDH antibody (It2006; red) (A) or a commercially available IMPDH antibody (IMPDH; red) (B) or both (commercially available IMPDH in green; It2006 in red) (C) are shown. In figures S2A and B, cells were coimmunolabeled for alpha-tubulin (green) and nuclei have been counterstained with DAPI (blue). Although a marked increase in RR number can be seen following ribavirin exposure, loukoumasome numbers were not increased. In spite of the prevalence of RR, with the exception of one cell, loukoumasomes immunoreactive for tubulin antibodies were not observed in this cell line (data not shown). D. A 24 hour exposure to 1 and 2 mM ribavirin (an IMPDH inhibitor) increased the number of R28 cells with RR but not retinal-loukoumasomes. RR were immunolabeled with anti-RR serum (It2006) and retinal-loukoumasomes were detected using an alpha-tubulin antibody. Data are represented as the mean ± SEM from 3 independent experiments. * p < 0.01, different from control. Significance was determined using an ANOVA and Fisher’s (LSD) test.
(TIF)

S3 Fig Detyrosinated-tubulin and MAP2 does not coimmunolabel neuronal-loukoumasomes in the rat pelvic ganglion. Neuronal loukoumasomes are clearly found within the autonomic neurons of the rat pelvic ganglion and are immunolabeled with the beta-III tubulin antibody SDL.3D10 (A, D). When costained with detyrosinated-tubulin (B, Millipore AB3201 at 1:200) or MAP2 (E, abcam ab5392 at 1:1000) it is clear that these antibodies do not recognize the neuronal-loukoumasomes. Composite images are shown in C and F. Scale bars represent 20 μm.
(TIF)
