# Supplementary figures and images for: Loukoumasomes Are Distinct Subcellular Structures from Rods and Rings and Are Structurally Associated with MAP2 and the Nuclear Envelope in Retinal Cells
Source: PLoS One. 2016 Oct 31;11(10):e0165162. doi: 10.1371/journal.pone.0165162 (PMC5087950; doi:10.1371/journal.pone.0165162)

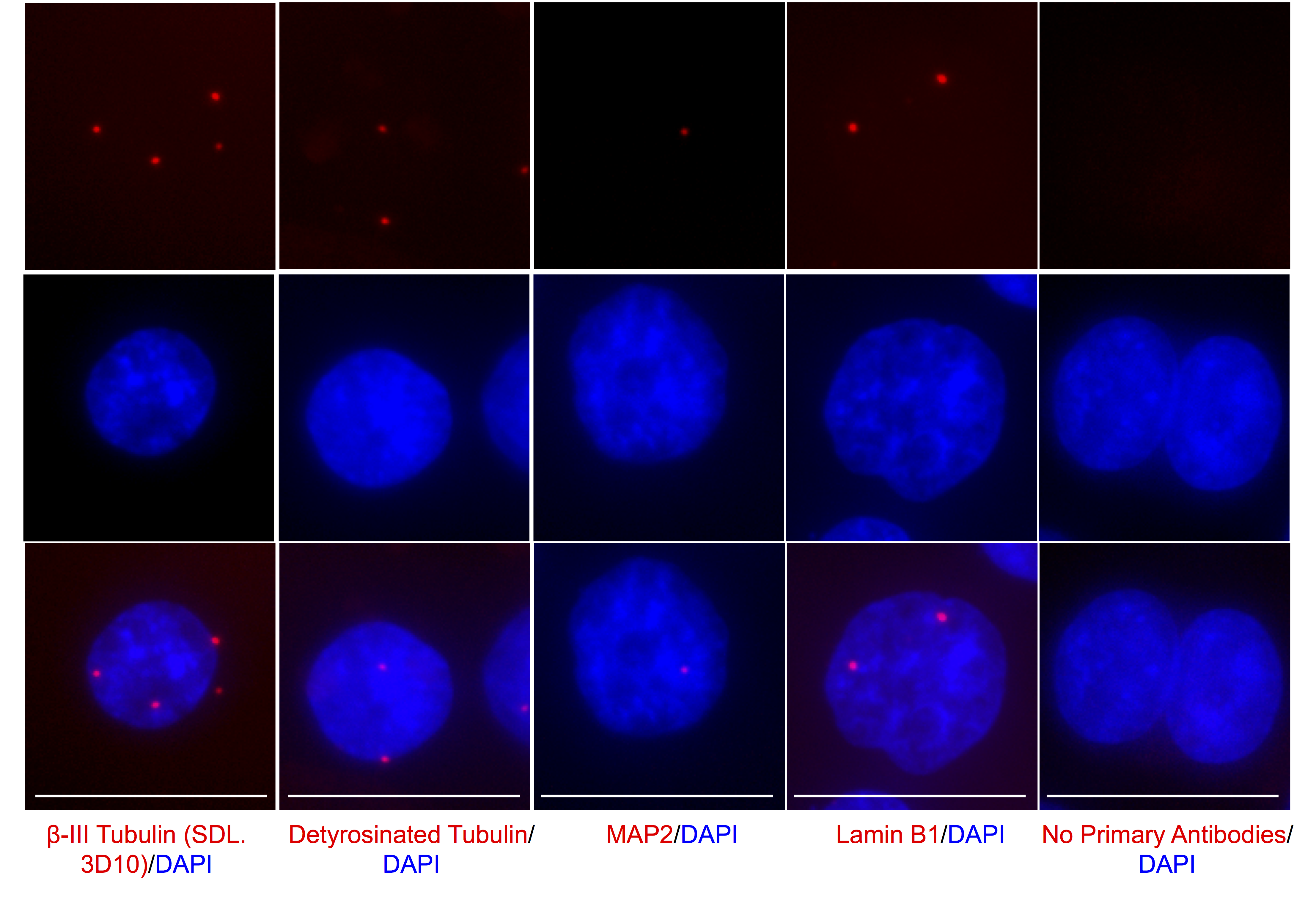

Supplement: S1 Fig — All antibodies used for the in situ PLA colocalization studies were also tested in single staining experiments. In Y79 cells, single primary antibodies were incubated together with both the anti-mouse and anti-rabbit PLA probes (see panels 1–4). Controls were also performed for these probes by omitting both primary antibodies in the incubations (see panel 5). (TIF) [file pone.0165162.s001.tif]

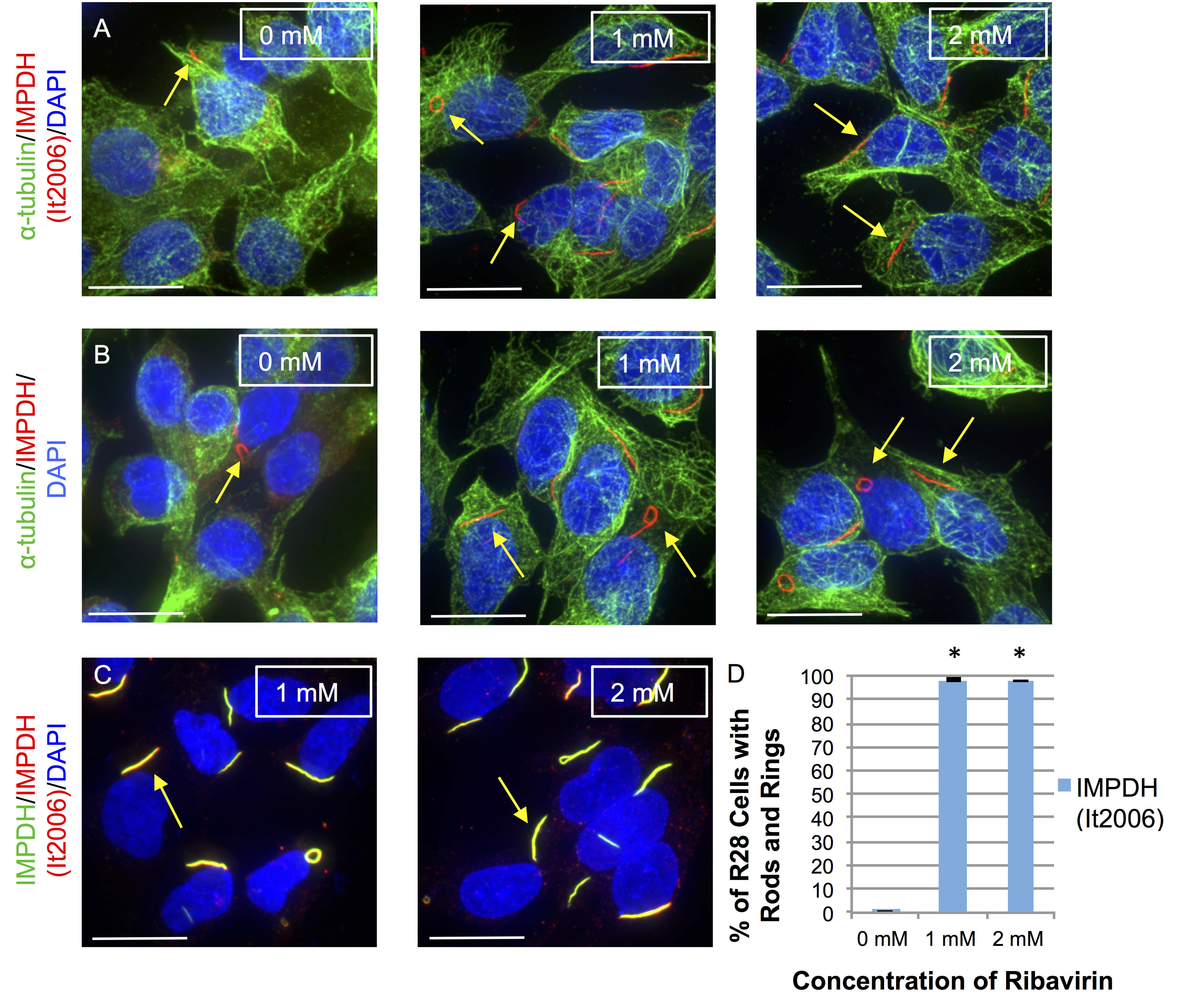

Supplement: S2 Fig — A-C. Representative composites of retinal R28 cells treated with ribavirin (1 mM or 2mM) or untreated control (0 mM) for 24 hours. Cells immunolabeled with either a human IMPDH antibody (It2006; red) (A) or a commercially available IMPDH antibody (IMPDH; red) (B) or both (commercially available IMPDH in green; It2006 in red) (C) are shown. In figures S2A and B, cells were coimmunolabeled for alpha-tubulin (green) and nuclei have been counterstained with DAPI (blue). Although a marked increase in RR number can be seen following ribavirin exposure, loukoumasome numbers were not increased. In spite of the prevalence of RR, with the exception of one cell, loukoumasomes immunoreactive for tubulin antibodies were not observed in this cell line (data not shown). D. A 24 hour exposure to 1 and 2 mM ribavirin (an IMPDH inhibitor) increased the number of R28 cells with RR but not retinal-loukoumasomes. RR were immunolabeled with anti-RR serum (It2006) and retinal-loukoumasomes were detected using an alpha-tubulin antibody. Data are represented as the mean ± SEM from 3 independent experiments. * p < 0.01, different from control. Significance was determined using an ANOVA and Fisher’s (LSD) test. (TIF) [file pone.0165162.s002.tif]

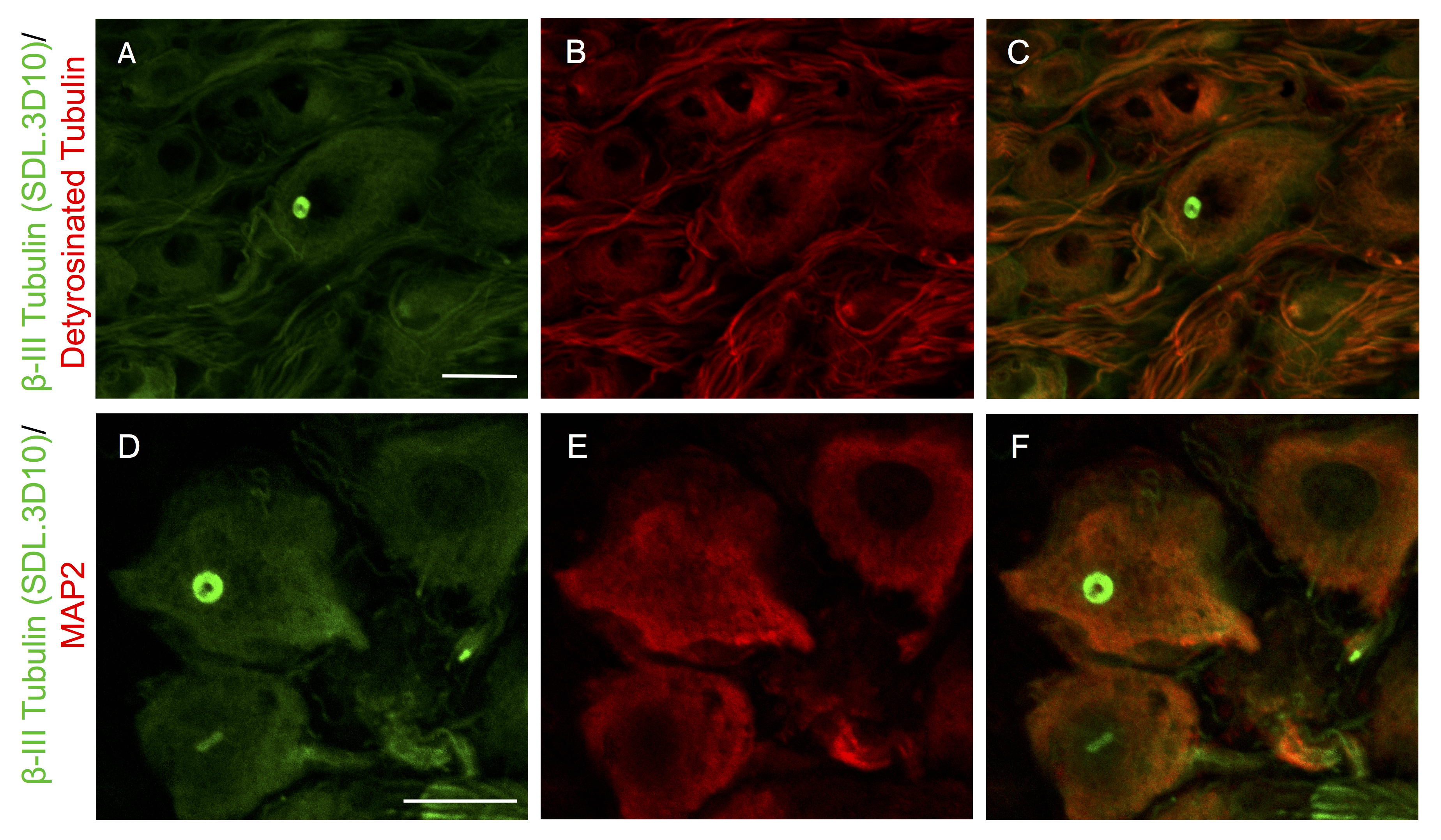

Supplement: S3 Fig — Neuronal loukoumasomes are clearly found within the autonomic neurons of the rat pelvic ganglion and are immunolabeled with the beta-III tubulin antibody SDL.3D10 (A, D). When costained with detyrosinated-tubulin (B, Millipore AB3201 at 1:200) or MAP2 (E, abcam ab5392 at 1:1000) it is clear that these antibodies do not recognize the neuronal-loukoumasomes. Composite images are shown in C and F. Scale bars represent 20 μm. (TIF) [file pone.0165162.s003.tif]
